# Profiles and Transitions of Sleep Disturbance and Depression Among Chinese Adolescents: The Predictive Roles of Life Stress and Resilience

**DOI:** 10.1155/da/3253107

**Published:** 2025-05-14

**Authors:** Dan Chen, Haoxian Ye, Luowei Bu, Wenxu Liu, Dongfang Wang, Fang Fan

**Affiliations:** ^1^School of Psychology, Centre for Studies of Psychological Applications, Guangdong Key Laboratory of Mental Health and Cognitive Science, Ministry of Education Key Laboratory of Brain Cognition and Educational Science, Guangdong Emergency Response Technology Research Center for Psychological Assistance in Emergencies, South China Normal University, Guangzhou, China; ^2^School of Management, Tianjin University of Traditional Chinese Medicine, Tianjin, China

**Keywords:** depression, life stress, profile, resilience, sleep disturbance, transition

## Abstract

**Purposes:** Sleep disturbance and depression co-occur frequently, yet their co-occurring and transitional nature among adolescents remains underexplored. Meanwhile, few studies have examined the potential predictive effect of environmental factors (e.g., life stress) and individual factors (e.g., resilience) on their interactive profiles and transitions. This study investigated the profiles and transitions of sleep disturbance and depression for Chinese adolescents, along with the predictive role of life stress and resilience in profiles and transitions.

**Methods:** A total of 17,404 adolescents (*M*_age_ = 12.1 ± 1.2 years, ranging from 10 to 17 years; 48.4% of females) were assessed at baseline from April 21 to May 12, 2021 (Time 1, T1), 6 months later from December 17–26, 2021 (Time 2, T2), and 1 year later from May 17 to June 6, 2022 (Time 3, T3). We used latent profile and latent transition analysis (LTA) to explore sleep disturbance and depression profiles and their transitions over time. Multivariate logistic regression was conducted to prove the predictive roles of stress and resilience in these profiles and transitions.

**Results:** Across all three time points, three profiles were consistently identified: low profile, co-occurring moderate profile, and co-occurring high profile. Three profiles presented distinct transition patterns, with adolescents in co-occurring high profiles displaying the highest level of transitions. The logistic regression suggested that adolescents with more interpersonal and academic stress or less resilience were more likely to belong to or transition into at-risk profiles.

**Conclusion:** Recognizing subgroup differences is crucial to understanding the co-occurrence and transitions of sleep disturbance and depression. Stress and resilience, particularly interpersonal stress, are significant predictors. This underscores the need importance for dynamically monitoring changes in sleep disturbance and depression, as well as identifying resilience and stress factors, which are essential for developing intervention programs.

**Summary**

• A large-sample, three-wave longitudinal study was conducted among Chinese adolescents.
• Sleep disturbance and depression exhibit both co-occurrence and heterogeneity.
• Three profiles demonstrate distinct patterns of stability and transitions.
• Interpersonal and academic stress are risk factors for profiles and transitions.
• Resilience serves as a protective factor for profiles and transition patterns.

## 1. Introduction

Adolescence is a vulnerable period for depression and sleep disturbance. The systematic review showed that globally, 37% of adolescents experience sleep disturbances [1], and 45% suffer from depression [2], rates higher than that observed in other age groups. In China, these rates are 26% [3] and 22% [4], respectively. Notably, both often co-occur, with up to 73% of adolescents with depression also experiencing sleep disturbance [5]. This co-occurrence is associated with an increased risk of relapse [6] and suicide [7] compared to the presence of either symptom alone. Thus, exploring the nature of the co-occurrence of depression and sleep disturbance, as well as identifying potential risk and protective factors, is crucial for promoting adolescent mental health.

The co-occurrence of depression and sleep disturbance is extensively documented. Individuals with sleep disturbance were over eight times more likely to develop depression than those without [8]. A prospective longitudinal study across eight waves in adolescents aged 10–12 years indicated significant bidirectional associations between depression and sleep disturbance and vice versa [9]. The majority of existing research focused on variable-centered approaches to demonstrate the co-occurrence of both. Individuals with co-occurrence also exhibit substantial heterogeneity; however, few person-centered studies have explored co-occurrence and its heterogeneity, such as using latent profile analysis (LPA). Furthermore, while variable-centered studies have demonstrated a strong overall association between sleep disturbance and depression, it remained unknown whether individuals with high levels of sleep disturbance also exhibit high levels of depression. Clarifying this relationship could provide deeper insight into the underlying nature of their co-occurrence [10].

Individuals were heterogeneous and underwent developmental changes, potentially entering or exiting certain profiles over time [11]. Latent transition analysis (LTA) extends LPA into a longitudinal framework, offering insights into continuity and transition patterns across profiles. Few studies have employed LTA to explore the dynamics of sleep disturbance and depression. For example, the only study investigating the stability and transition of sleep disturbance among 10,313 adolescents indicated that adolescents in low sleep disturbance profiles showed relatively high stability (77%), whereas those in high disturbance profiles showed comparatively low stability [12]. In addition, Herman et al. identified three depression profiles, namely low profile, moderated profile, and high profile. Concretely speaking, individuals in the low profile maintained high stability (67.5%), whereas those in the high profile transitioned to the low profile (67%), followed by the moderated profile (28%) [13]. However, exploring the transitions of sleep disturbance or depression separately may be limited and insufficient, especially given the shared bio-psycho-social vulnerabilities underlying both issues. Therefore, it was necessary to examine both variables to explore their transition patterns, which may offer novel insights into their potential joint changes.

The diathesis-stress model suggests that mental health results from the interaction between vulnerability (e.g., low resilience) and external stressors [14]. Especially, perceived stress is a well-documented trigger for adolescent psychopathology [15], while resilience serves as a protective factor that mitigates its adverse effects [16]. Examining both factors enables a more comprehensive understanding of the real situation adolescents actually face. Longitudinal studies also have shown that resilience predicts both sleep disturbance [17] and depression [18], highlighting it as a key modifiable protective factor. Additionally, adolescents primarily face interpersonal and academic stress, which are strongly linked to sleep disturbance [19, 20] and depression [21, 22]. However, these studies used a variable-centered approach, which ignored individual differences in developmental changes. For instance, although resilience was linked to depression and sleep disturbance, it remains uncertain whether adolescents in the high depression-sleep disturbance profile could transition to the low profile due to their high resilience. Clarifying this could further illuminate the mechanism underlying the co-occurrence and transition between depression and sleep disturbance.

Prior studies have supported that sleep disturbance and depression are associated with family socioeconomic status (SES), gender, and age [23], making it methodologically critical to adjust for these factors to independently evaluate predictive relationships. Additionally, given the limited understanding of sleep disturbance and depression profiles, their transition patterns, and the roles of interpersonal stress, academic stress, and resilience in these dynamics, this study aims to (1) identify sleep disturbance-depression profiles using LPA across three time points; (2) examine transitions between profiles using LTA, with the expectation that the low-risk profile might remain the greatest stability, whereas other profiles exhibit more transitions; and (3) explore the effects of interpersonal stress, academic stress, and resilience on profiles and transitions in Chinese adolescents. We hypothesize that higher levels of interpersonal and academic stress will increase the likelihood of being in or transitioning to at-risk profiles, while greater resilience will reduce this likelihood.

## 2. Methods

### 2.1. Participants

**Figure 1:**
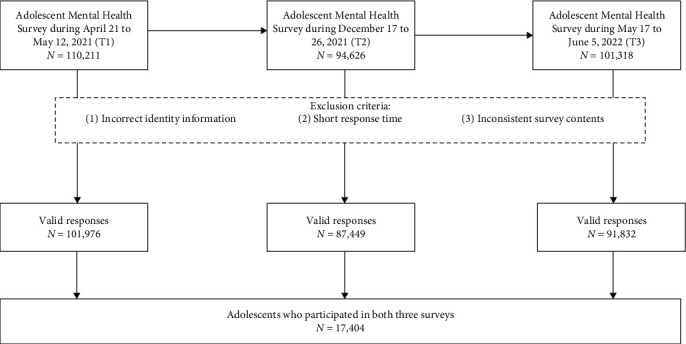
Participation flowchart.

Data were obtained from the Adolescent Mental Health Survey (AMHS) conducted in a district of Shenzhen City, Guangdong Province, China. The AMHS is routinely administered each academic semester by the local education bureau, adopting cluster convenience sampling in each round. As presented in Figure 1, the present study extracted three waves of surveys: from April 21 to May 12, 2021 (Time 1, T1); from December 17–26, 2021 (Time 2, T2); and from May 17 to June 6, 2022 (Time 3, T3). At T1, the study initially targeted a sample of 110,211 students in the 5th, 6th (i.e., the fifth and sixth year of primary school), 7th, 8th, and 9th grades (i.e., the first, second, and third year of junior high school). It should be noted that 8th students did not participate in T2 surveys due to upcoming entrance exams. The number of students was 94,626 at T2 and 101,318 at T3. The data collection method employed in this study has been previously validated and utilized in several publications [24, 25], confirming its reliability and validity. Each student was assigned a unique ID for identification purposes. The survey system incorporated automatic completeness recognition and limits on the number of entries, and so on. Following the survey, we implemented data quality control measures. Especially, participants were excluded based on criteria, including (1) incorrect identity information (e.g., incorrectly filled in student number; student number occur more than once), (2) short response times (e.g., less than 5 min), and/or (3) inconsistent survey contents (e.g., regular answers). A small number of samples were excluded because they met the predefined exclusion criteria, leaving 101,976, 87,449, and 91,832 students in the T1, T2, and T3 samples, respectively. Each student in the school is assigned a unique and permanent student number. To ensure the accuracy and consistency of our data, we utilized a precise matching approach based on students' school, class, and student number to identify those who participated in all three waves of the survey. This rigorous matching process enabled the tracking of individual participants across the three time points and facilitated the exclusion of incomplete or inconsistent entries. Ultimately, a total of 17,404 students who completed all three surveys were included in the final dataset for analysis in this study (Figure 1).

This research adopted a repeated cross-sectional design across three time points, incorporating a nested longitudinal subsample. This study was conducted using the “Survey Star” online system. With the local education bureau's assistance, school psychology teachers distributed survey invitations to students. Participants and their guardians completed an electronic informed consent form before the survey. Subsequently, students who consented to participate accessed the web-based questionnaire by scanning its quick response (QR) code using either computers or mobile phones. All data were confidential, and students could retain the freedom to withdraw at any time. The investigation adhered to the latest principles of the Helsinki Declaration and received approval from the Ethics Committees of South China Normal University.

### 2.2. Measures

#### 2.2.1. Depression

The Chinese edition of the Patient Health Questionnaire-9 (PHQ-9) was used to measure depression symptoms in the past 2 weeks [26], showing robust-to-satisfactory psychometric properties. Participants were asked to respond on a 4-point Likert scale, ranging from 1 (not at all) to 4 (nearly every day). A higher total score indicates a higher level of depression symptoms. In the present study, the Cronbach's *α* reached 0.90, 0.91, and 0.92 at T1, T2, and T3, respectively.

#### 2.2.2. Sleep Disturbance

The Chinese version of sleep disturbance was assessed by a subset of four items deriving from the Youth Self-Rating Insomnia Scale (YSIS) [27] in the past month, including difficulty initiating sleep (DIS), difficulty maintaining sleep (DMS), early morning awakening (EMA) and overall sleep quality. Participants rated four experiences on a 5-point Likert scale. The responses to DIS, DMS, and EMA included: never, <1 time/week, 1–2 times/week, 3–5 times/week, and 6–7 times/week. Participants who responded with “3–5 nights/week” or “6–7 nights/week” to any of the three questions were classified as exhibiting insomnia symptoms. The sleep quality answers included: very good, good, fair, poor, and very poor. We classified participants who answered “poor” or “very poor” as having poor sleep quality. The severity of sleep disturbance was assessed by calculating the total score of four items, following the previous study [28]. The scale demonstrated excellent internal consistency in this study, with Cronbach's *α* of 0.81 at T1 and 0.82 at T2 and T3.

#### 2.2.3. Life Stress

Academic and interpersonal stresses were measured by two dimensions of the Chinese version of the Adolescent Self-Rating Life Events Checklist (ASLEC) [29], which has shown satisfactory reliability and validity among Chinese adolescents [24]. The ASLEC incorporates 27 items, including six dimensions: interpersonal relationships, academic stress, being punished, bereavement, change for adaptation, and others. The dimensions of academic stress and interpersonal stress each include five items. Participants rated each item on a 5-point scale, indicating from 1 “not at all” to 5 “extremely severe,” to assess the stresses they have perceived over the last 6 months (i.e., from T1 to T2, from T2 to T3). The items were aggregated to produce a total score, with higher scores indicating greater severity of perceived stresses. In the current study, Cronbach's *α* for academic and interpersonal stress were 0.87 and 0.88, respectively, between T1 and T2, and 0.85 and 0.85, between T2 and T3.

#### 2.2.4. Resilience

The Chinese version of the Connor–Davidson Resilience Scale-10 item (CD-RISC-10) evaluated resilience [30], which exhibited good reliability and validity. The responses to each item ranged from 0 (“not true at all”) to 4 (“true nearly all the time”), and were summed to generate a total score, with higher scores indicating greater resilience. The current study yielded Cronbach's *α* values of 0.95 at T1 and 0.97 at T2.

#### 2.2.5. Covariates

At T1, the student's age, gender (1 = boys; 2 = girls), and SES were assessed. Parental occupations, educational attainment, and monthly family income were used as the indicators of SES. Students reported on the fathers' and mothers' education levels (1 = never attended school to 7 = master degree), occupational status (1 = have no stable work, 2 = have stable work), and monthly family income (1 = < 6,000 RMB, 8 = > 42,000 RMB). Scores for five indicators were standardized, and the average was calculated based on the five standardized variables to index SES [22].

### 2.3. Data Analysis

First, the Kolmogorov–Smirnov tests indicated that all continuous variables deviated significantly from normality (*p*  < 0.001). Given the nonnormality of the data, Spearman correlation analyses were employed using SPSS 26.0 to examine the relationships among the study variables. This method is chosen over Pearson's correlation, as it does not require the assumption of normality and is more robust to skewed distributions [31]. Second, LPAs were performed with Mplus 8.3 to identify profiles. To facilitate interpretation, all variables were standardized into *Z*-scores before conducting the LPAs, which were estimated for solutions ranging from two to five profiles. Model fit was evaluated using the following criteria: (1) low values of Akaike information criterion (AIC), Bayesian information criterion (BIC), and sample-size adjusted BIC (A-BIC); (2) high entropy (>0.80); (3) high average latent class posterior probabilities (>0.70); (4) significant results on the Lo–Mendel–Rubin likelihood ratio test (LMR-LRT) and (5) sufficient sample size for each profile (>1%) [32]. The interpretability of the profiles, specifically whether the addition of another profile revealed a qualitatively distinct profile, was also considered. Third, LTA was utilized to examine transitions between profiles over time (from T1 to T2, T1 to T3, and T2 to T3). Finally, multinomial logistic regression analysis, using the manual 3-step approach [33], was applied to assess the effects of academic stress, interpersonal stress, and resilience on profiles and their transitions. Gender, age, and SES were included as covariates in the analyses.

## 3. Results

### 3.1. Descriptive Statistics

A total of 17,404 students were included in the final analyses, comprising 8986 males (51.6%) and 8418 females (48.4%). The mean age of the sample was 12.11 years (SD = 1.22), with an age range of 10–17 years. Additionally, the Spearman correlations for the continuous variables and the point-biserial correlation for the relationship between gender and continuous variables at three waves are provided in Table 1.

### 3.2. LPA

**Figure 2:**
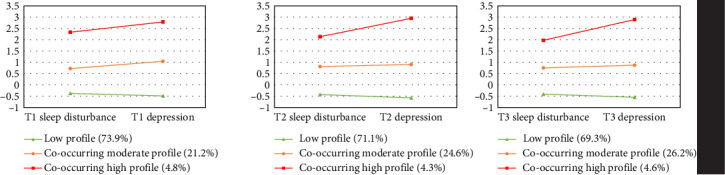
Profiles of sleep disturbance and depression across three times.

LPA was conducted at three time points separately. The fit indices for LPA are presented in Table 2. The three-profile model was identified as the most suitable across the three time points based on the following evidence: the largest drop in AIC, BIC, and A-BIC was observed between the two-profile and three-profile models, indicating the superior fit of the three-profile model. For the three-profile model, not only was the LMR-LRT statistically significant, but the entropy also satisfied the recommended threshold. The four-profile and five-profile models were excluded due to the inclusion of a class with a small sample size (<3%). Additionally, compared to the interpretability of the three profiles, the four-profile model failed to reveal a qualitatively distinct profile. Based on the standard mean of depression and sleep disturbance, the three profiles at the three time points were labeled as follows: low profile (low level of both sleep disturbance and depression), co-occurring moderate profile (moderate level of both sleep disturbance and depression), and co-occurring high profile (high level of both). Details for each profile can be found in Figure 2.

### 3.3. LTA

LTA was used to assess profile stability and transitions.  Table 3 shows the transition probabilities from T1 to T2, T2 to T3, and T1 to T3. The low profile had the highest stability across all time points over the 6-month period, followed by the co-occurring moderate and co-occurring high profiles. For those who exhibited instability across three transitions, adolescents initially in the low profile primarily transitioned to the co-occurring moderate profile, with very few shifting to the co-occurring high profile. Those in the co-occurring moderate profile tended to change to the low profile, with only a small number transitioning to the co-occurring high profile. It is noteworthy that adolescents in co-occurring high profiles also showed high stability, with possibilities of 36.9%, 46.6%, and 32.1% across the respective time points. Some of them transitioned into the co-occurring moderate profile, followed by the low profile.

### 3.4. Predictors of Profiles and Transitions

This study employed multinomial logistic regressions to assess the influence of resilience and interpersonal and academic stress on depression and sleep disturbance profiles and transitions, with age, gender, and SES as control variables.  Table 4 shows the odds ratios (ORs) for how factors at T1 predicted profiles across three time points.  Table 5 shows the ORs for how factors at T2 predicted profiles at T2 and T3. Adolescents with higher levels of interpersonal and academic stress were more likely to belong to the co-occurring moderate and high profiles across all three time points. Conversely, those with greater resilience were less likely to be classified into these profiles.

The results of the profile transitions are shown in Tables 6–8. Adolescents in low profiles with higher levels of interpersonal and academic stress at T1 or T2 tended to transition to the co-occurring moderate and high profiles than to remain in this profile. Adolescents in the co-occurring high profile at T1 or T2 suffering from more interpersonal stress tended to stay in this profile over time instead of transitioning to the co-occurring moderate and low profile. Adolescents in the co-occurring moderate profiles at T1 or T2 suffering from higher levels of interpersonal and academic stress tended to transition to the co-occurring high profile over time rather than staying in this profile or transitioning to the low profile. Conversely, adolescents in low profiles with greater resilience at T1 or T2 were less likely to transition to the co-occurring moderate profile than to remain in this profile. Adolescents in co-occurring moderate profiles experiencing greater resilience at T1 or T2 were less likely to transition to the co-occurring high profiles than remaining in this profile. Additionally, adolescents in co-occurring high profiles with greater resilience at T1 or T2 were inclined to transition to the co-occurring moderate profiles rather than staying in the high profile.

## 4. Discussions

Drawing on a person-centered approach, this study sought to increase the understanding of subgroup differences and transitions between the two phenomena, as well as the role of stress and resilience in shaping these profiles and transitions, thereby providing further empirical evidence for understanding their comorbidity. Our key findings were summarized as follows: (1) the co-occurrence of sleep disturbance and depression exhibited heterogeneity and could be categorized into three distinct profiles; (2) individuals within these three profiles demonstrated distinct patterns of stability and transition; (3) academic stress and interpersonal stress were risk factors for profile memberships and transition patterns. Compared to academic stress, interpersonal stress was more strongly associated with an increased likelihood of individuals belonging to or transitioning into moderate or high profiles; (4) resilience served as a protective factor for profile memberships and transition patterns.

Our study provided new empirical support for the co-occurrence of sleep disturbance and depression. We speculated that this might be attributed to shared common susceptibility risk factors, such as intrafamily conflict [34], bullying victimization [35], and reduced serotonin [36]. In addition, sleep disturbance and depression were heterogeneous and could be classified into three profiles, which were consistent with previous studies. Previously, Chen et al. [37] demonstrated that using LPA, three distinct profiles of sleep disturbance were observed among 4302 college students: high profile (10.6%), mild profile (37.5%), and no sleep disturbance profile (51.9%). Additionally, Sabiston et al. [38] identified three depression trajectories in a sample of 860 adolescents: low and declining group (37.8%), moderate and stable group (41.6%), and high increasing group (20.6%). Notably, this study identified at-risk profiles (moderate and high profiles) characterized by consistently higher levels of sleep disturbance and depression than the low profile. The results might stem from substantial biological, cognitive, socioemotional, and behavioral changes during early adolescence, such as shifts in daily routines (e.g., later bedtimes and earlier wake-up times). Some individuals failed to adapt to these changes, adversely affecting their sleep and mental health [39].

Regardless of the classification period from T1 to T2, T2 to T3, or T1 to T3, most students in the low profile remained stable over time (82.6%−86.7%). In addition, the co-occurring moderate profile (53.0%−63.3%) showed relatively high stability, while the co-occurring high profile (32.1%−46.6%) showed the lowest stability. This finding is consistent with previous studies [40]. For transitions, this study found that high-profile adolescents had the highest likelihood of transitioning to the moderate profile over time (37.4%−44.0%), followed by the low profile (16.0%−23.9%). Co-occurring moderate profiles also tended to transition to the low profile (30.0%−39.0%), with fewer transitioning to the high profile (6.7%−8.0%). Low-profile students were more likely to transition to the moderate profile (12.1%−15.8%) than to the high profile (1.2%−1.6%). These findings align with previous studies concerning sleep disturbance transitions, which revealed similar patterns: high profile transitioning to moderate profile, low profile to moderate profile, and moderate profile to low profile [12]. Similarly, research on depression transitions indicated that individuals in high profile often shifted to low profile, moderate profile to low profile, and low profile to moderate profile [13, 41]. Overall, adolescence is marked by fluid mental health statuses, with adolescents more likely to display a “recovery” pattern—progressing toward the low profile rather than regressing toward the high profile over time. This pattern aligned with existing research on co-occurring depression and non-suicidal self-injury, as well as co-occurring depression and loneliness [10, 23]. This also might be explained by adolescents' improved ability to cope with sleep disturbance and depression due to their increased adaptability and emotional skills in the long run [42]. However, individuals who either remained in the high profile or transitioned to the high profile over time may be at potential risk and require external assistance for improvement. For example, implementing a combined approach of cognitive behavior therapy for adolescent depression (CBT-D) with CBT for insomnia (CBT-I) has been documented to have medium-to-large effects on improving sleep and reducing depression [43].

Research indicated that both trainable internal factors (resilience) and improvable external factors (interpersonal and academic stress) can interact to influence sleep disturbances and depression. Specifically, adolescents who perceived more interpersonal or academic stress or less resilience were more likely to be classified into or transition to a high-risk profile rather than a low profile. On the one hand, during adolescence, the pursuit of interpersonal relationships and meaningful social–emotional interactions intensifies, making individuals more vulnerable to the negative effects of problematic interpersonal relationships [44]. According to the interpersonal risk model, this increased the risk of developing depression and sleep disturbance [45, 46]. In addition, upon entering adolescence, academic achievement gradually became a central focus. The pressure to maintain high academic performance, coupled with widespread academic stress, may lead individuals to sacrifice sleep time in favor of studying [47] while also diminishing cognitive resources to effectively regulate their emotions [48]. On the other hand, this result supported that resilience can be viewed as a defense mechanism, aiding individuals in maintaining mental well-being in the face of adversity [49]. A possible explanation was that resilience was closely linked to a range of positive psychological factors, such as positive cognition style [50] and emotion regulation strategy [51], which were beneficial to the development of sleep disturbance and depression [52, 53]. Furthermore, interpersonal stress manifested more stable and robust effects on profiles and transitions compared to academic stress. Therefore, it is particularly important to implement targeted interventions for interpersonal stress, such as “social and emotional learning” [54], which includes various prevention programs designed to foster social skills. The finding emphasized the necessity of timely monitoring and adjustment of the external stress faced by adolescents, as well as the importance of cultivating resilience as an internal resource to reduce individual susceptibility to psychopathology.

Despite its strengths, including a large adolescent sample and a longitudinal design, several limitations should be acknowledged. First, the sample was drawn from a single region, which may limit the generalizability of the results. Future research needs to validate these results in other regions of Chinese or in different countries. Second, the longitudinal design of the three time points spanned only one and a half years, which may limit the detection of meaningful findings. Future studies could span the entire adolescence. Third, the study used self-report questionnaires instead of clinical diagnoses and objective physiological indicators, which may lead to reporting bias and affect validity. Future research should incorporate objective measures and clinical interviews to evaluate the intensity of sleep disturbance and depression. Fourth, the use of QR codes may still pose limitations in ensuring respondent authenticity. Future studies could adopt stricter measures, such as facial recognition technology, to further enhance authenticity. Finally, the longitudinal survey exhibited an attrition rate, potentially influenced by several factors: (1) absence of senior students due to high-stakes examinations (e.g., college entrance exams), (2) delayed survey notification to participating schools, and (3) scheduling conflicts with institutional priorities (e.g., athletic meets, pre-scheduled midterm tests) that prevented survey administration. These limitations may compromise the accuracy of symptom change measurements, necessitating cautious interpretation of results.

Thus, it is imperative for both school professionals and parents to recognize that differentiated mental health promotion and intervention programs are likely to be more effective for specific subgroups of adolescents with distinct characteristics. Simultaneously, monitoring resilience, stress, sleep disturbances, and depressive symptoms is crucial to detect the status of mental health during this developmental stage, guaranteeing timely interventions. Furthermore, to enhance resilience and mitigate academic and interpersonal stress, educators and parents should prioritize the integration of evidence-based social and emotional learning programs [54] and resilience-building interventions (e.g., mindfulness and attention training) [55] into both classroom curricula and home-based activities. Most importantly, it is essential to reduce academic pressure, as students' mental and physical well-being should be prioritized above academic achievement.

## 5. Conclusions

In conclusion, through the person-centered approach, our study identified three profiles of sleep disturbance and depression with distinct transition patterns, as well as explored the role of resilience and interpersonal and academic stress. Especially, adolescents in low profile indicated a stable pattern; however, those in at-risk profiles showed varying transition patterns. Adolescents with more stress and less resilience tend to belong to and transition to an at-risk profile. These findings underscore the heterogeneity in the progression of sleep disturbances and depression, thereby challenging the applicability of a “one-size-fits-all” intervention approach. It is imperative to develop tailored strategies for distinct subgroups, including timely monitoring and targeted support for individuals with deteriorating patterns. Educational institutions should implement comprehensive stress management programs, such as flexible curriculum design and social–emotional learning programs. Concurrently, parents should prioritize fostering resilience and interpersonal skills in their children from an early age, rather than focusing solely on academic achievement, to mitigate the risk of depression and sleep disturbances. Furthermore, the generalizability of these findings necessitates further validation through studies involving diverse age samples and cultural contexts. Future research should employ more rigorous data collection procedures, investigate additional risk and protective factors, and extend the longitudinal timeframe to encompass the entire adolescent period.

## Figures and Tables

**Table 1:** Bivariate correlations among variables.

Variables	1	2	3	4	5	6	7	8	9	10	11	12	13	14	15
1 T1SD	1	—	—	—	—	—	—	—	—	—	—	—	—	—	—
2 T1 DE	0.59*⁣*^*∗∗*^	1	—	—	—	—	—	—	—	—	—	—	—	—	—
3 T2 SD	0.41*⁣*^*∗∗*^	0.37*⁣*^*∗∗*^	1	—	—	—	—	—	—	—	—	—	—	—	—
4 T2 DE	0.34*⁣*^*∗∗*^	0.46*⁣*^*∗∗*^	0.62*⁣*^*∗∗*^	1	—	—	—	—	—	—	—	—	—	—	—
5 T3 SD	0.39*⁣*^*∗∗*^	0.34*⁣*^*∗∗*^	0.51*⁣*^*∗∗*^	0.42*⁣*^*∗∗*^	1	—	—	—	—	—	—	—	—	—	—
6 T3 DE	0.31*⁣*^*∗∗*^	0.41*⁣*^*∗∗*^	0.42*⁣*^*∗∗*^	0.53*⁣*^*∗∗*^	0.63*⁣*^*∗∗*^	1	—	—	—	—	—	—	—	—	—
7 T1 IS	0.23*⁣*^*∗∗*^	0.30*⁣*^*∗∗*^	0.38*⁣*^*∗∗*^	0.51*⁣*^*∗∗*^	0.29*⁣*^*∗∗*^	0.37*⁣*^*∗∗*^	1	—	—	—	—	—	—	—	—
8 T1 AS	0.21*⁣*^*∗∗*^	0.28*⁣*^*∗∗*^	0.36*⁣*^*∗∗*^	0.48*⁣*^*∗∗*^	0.27*⁣*^*∗∗*^	0.35*⁣*^*∗∗*^	0.83*⁣*^*∗∗*^	1	—	—	—	—	—	—	—
9 T1 RE	−0.30*⁣*^*∗∗*^	−0.42*⁣*^*∗∗*^	−0.22*⁣*^*∗∗*^	−0.28*⁣*^*∗∗*^	−0.21*⁣*^*∗∗*^	−0.25*⁣*^*∗∗*^	−0.21*⁣*^*∗∗*^	−0.19*⁣*^*∗∗*^	1	—	—	—	—	—	—
10 T2 IS	0.22*⁣*^*∗∗*^	0.28*⁣*^*∗∗*^	0.31*⁣*^*∗∗*^	0.38*⁣*^*∗∗*^	0.40*⁣*^*∗∗*^	0.53*⁣*^*∗∗*^	0.47*⁣*^*∗∗*^	0.44*⁣*^*∗∗*^	−0.19*⁣*^*∗∗*^	1	—	—	—	—	—
11 T2 AS	0.20*⁣*^*∗∗*^	0.26*⁣*^*∗∗*^	0.28*⁣*^*∗∗*^	0.35*⁣*^*∗∗*^	0.37*⁣*^*∗∗*^	0.51*⁣*^*∗∗*^	0.43*⁣*^*∗∗*^	0.48*⁣*^*∗∗*^	−0.17*⁣*^*∗∗*^	0.83*⁣*^*∗∗*^	1	—	—	—	—
12 T2 RE	−0.21*⁣*^*∗∗*^	−0.28*⁣*^*∗∗*^	−0.35*⁣*^*∗∗*^	−0.45*⁣*^*∗∗*^	−0.28*⁣*^*∗∗*^	−0.33*⁣*^*∗∗*^	−0.35*⁣*^*∗∗*^	−0.34*⁣*^*∗∗*^	0.42*⁣*^*∗∗*^	−0.28*⁣*^*∗∗*^	−0.27*⁣*^*∗∗*^	1	—	—	—
13 T1 Age	0.10*⁣*^*∗∗*^	0.13*⁣*^*∗∗*^	0.15*⁣*^*∗∗*^	0.18*⁣*^*∗∗*^	0.17*⁣*^*∗∗*^	0.20*⁣*^*∗∗*^	0.14*⁣*^*∗∗*^	0.17*⁣*^*∗∗*^	−0.03*⁣*^*∗∗*^	0.26*⁣*^*∗∗*^	0.26*⁣*^*∗∗*^	−0.13*⁣*^*∗∗*^	1	—	—
14 T1 Gender	0.07*⁣*^*∗∗*^	0.12*⁣*^*∗∗*^	0.10*⁣*^*∗∗*^	−0.14*⁣*^*∗∗*^	0.12*⁣*^*∗∗*^	0.16*⁣*^*∗∗*^	0.12*⁣*^*∗∗*^	0.12*⁣*^*∗∗*^	−0.12*⁣*^*∗∗*^	0.12*⁣*^*∗∗*^	0.13*⁣*^*∗∗*^	−0.13*⁣*^*∗∗*^	0.00	1	—
15 T1 SES	0.00	0.00	0.00	0.00	0.00	0.00	0.00	−0.03*⁣*^*∗∗*^	0.12*⁣*^*∗∗*^	−0.03*⁣*^*∗∗*^	−006*⁣*^*∗∗*^	0.08*⁣*^*∗∗*^	−0.07*⁣*^*∗∗*^	0.00	1

Abbreviations: AS, academic stress; DE, depression; IS, interpersonal stress; RE, resilience; SD, sleep disturbances; T1, Time 1; T2, Time 2; T3, Time 3.

*⁣* ^*∗*^ *p* < 0.01; *⁣*^*∗∗*^*p* < 0.001.

**Table 2:** Model fit indices for latent profile analyses in Time 1, Time 2, and Time 3.

	Classes	AIC	BIC	A-BIC	Entropy	LRT	Smallest profile size
Time 1	2-profile	86,526.416	86,580.767	86,558.522	0.903	11,861.488*⁣*^*∗∗*^	14.8%
	**3-profile**	**82,273.368**	**82,351.013**	**82,319.233**	**0.903**	**4118.455** *⁣* ^*∗∗*^	**4.8%**
	4-profile	79,738.808	79,839.746	79,798.433	0.915	2456.695*⁣*^*∗∗*^	2.4%
	5-profile	78,033.298	78,157.529	78106.682	0.913	1655.013*⁣*^*∗∗*^	2.2%

Time 2	2-profile	86,309.096	86,363.447	86,341.201	0.893	12,071.635*⁣*^*∗∗*^	17.0%
	**3-profile**	**82,125.064**	**82,202.709**	**82,170.929**	**0.896**	**4051.717** *⁣* ^*∗∗*^	**4.3%**
	4-profile	79,932.434	80,033.372	79,992.059	0.910	2126.052*⁣*^*∗∗*^	1.8%
	5-profile	78,456.886	78,581.118	78,530.271	0.881	1432.641*⁣*^*∗∗*^	2.2%

Time 3	2-profile	86,737.524	86,791.875	86,769.629	0.877	11,657.349*⁣*^*∗∗*^	18.3%
	**3-profile**	**81,590.843**	**81,668.487**	**81,636.708**	**0.913**	**4982.588** *⁣* ^*∗∗*^	**4.6%**
	4-profile	79,523.892	79,624.830	79,583.517	0.928	2004.522*⁣*^*∗∗*^	1.7%
	5-profile	77,234.643	77,358.874	77,308.027	0.917	2219.482*⁣*^*∗∗*^	1.0%

*Note:* Bolded values indicate the preferred model for a given fit index.

Abbreviations: ABIC, adjusted Bayesian information criteria (sample size); AIC, Akaike information criteria; LRT, Lo–Mendell–Rubin likelihood test (adjusted).

*⁣* ^*∗*^ *p* < 0.01; *⁣*^*∗∗*^*p* < 0.001.

**Table 3:** Unconditional latent transition probabilities from T1 to T2, T2 to T3, and T1 to T3.

	Time 2			Time 3		
	Low profile	Co-occurring moderate profile	Co-occurring high profile	Low profile	Co-occurring moderate profile	Co-occurring high profile
Time 1						
Low profile	0.857	0.128	0.014	0.867	0.121	0.012
Co-occurring moderate profile	0.345	0.580	0.075	0.300	0.633	0.067
Co-occurring high profile	0.222	0.409	0.369	0.160	0.374	0.466
Time 2						
Low profile	—	—	—	0.826	0.158	0.016
Co-occurring moderate profile	—	—	—	0.390	0.530	0.080
Co-occurring high profile	—	—	—	0.239	0.440	0.321

**Table 4:** Multinomial logistic regression of T1 predictors on sleep disturbance and depression profiles at T1, T2, and T3.

T1 predictors	Reference class: Low profile (T1)				Reference class: Low profile (T2)				Reference class: Low profile (T3)			
	Co-occurring moderate profile		Co-occurring high profile		Co-occurring moderate profile		Co-occurring high profile		Co-occurring moderate profile		Co-occurring high profile	
	OR	95% CI	OR	95% CI	OR	95% CI	OR	95% CI	OR	95% CI	OR	95% CI
SES	1.14*⁣*^*∗∗∗*^	[1.07, 1.22]	1.17*⁣*^*∗*^	[1.03, 1.34]	1.13*⁣*^*∗∗∗*^	[1.05, 1.21]	1.14	[0.99, 1.32]	1.11*⁣*^*∗∗∗*^	[1.04, 1.19]	1.06	[0.93, 1.21]
Gender	1.20*⁣*^*∗∗∗*^	[1.15, 1.24]	1.61*⁣*^*∗∗∗*^	[1.48, 1.74]	1.15*⁣*^*∗∗∗*^	[1.11–, 1.20]	1.48*⁣*^*∗∗∗*^	[1.36, 1.61]	1.24*⁣*^*∗∗∗*^	[1.19, 1.28]	1.60*⁣*^*∗∗∗*^	[1.47, 1.73]
Age	1.37*⁣*^*∗∗∗*^	[1.32, 1.42]	1.44*⁣*^*∗∗∗*^	[1.34, 1.55]	1.41*⁣*^*∗∗∗*^	[1.36, 1.47]	1.49*⁣*^*∗∗∗*^	[1.38, 1.60]	1.48*⁣*^*∗∗∗*^	[1.43, 1.54]	1.51*⁣*^*∗∗∗*^	[1.41, 1.63]
T1 IS	—	—	—	—	1.92*⁣*^*∗∗∗*^	[1.81,2.05]	3.61*⁣*^*∗∗∗*^	[3.22, 4.05]	1.48*⁣*^*∗∗∗*^	[1.40, 1.58]	2.09*⁣*^*∗∗∗*^	[1.87, 2.32]
T1 AS	—	—	—	—	1.34*⁣*^*∗∗∗*^	[1.26, 1.42]	1.42*⁣*^*∗∗∗*^	[1.28, 1.58]	1.22*⁣*^*∗∗∗*^	[1.22, 1.15]	1.37*⁣*^*∗∗∗*^	[1.23, 1.52]
T1 RE	0.50*⁣*^*∗∗∗*^	[0.48, 0.52]	0.31*⁣*^*∗∗∗*^	[0.29, 0.33]	0.63*⁣*^*∗∗∗*^	[0.61, 0.66]	0.51*⁣*^*∗∗∗*^	[0.47, 0,55]	0.70*⁣*^*∗∗∗*^	[0.67, 0.73]	0.56*⁣*^*∗∗∗*^	[0.52, 0.60]

Abbreviations: AS, academic stress; IS, interpersonal stress; RE, resilience.

*⁣* ^*∗*^ *p* < 0.05; *⁣*^*∗∗*^*p* < 0.01; *⁣*^*∗∗∗*^*p* < 0.001.

**Table 5:** Multinomial logistic regression of T2 predictors on sleep disturbance and depression profiles at T2 and T3.

T2 predictors	Reference class: Low profile (T2)				Reference class: Low profile (T3)			
	Co-occurring moderate profile		Co-occurring high profile		Co-occurring moderate profile		Co-occurring high profile	
	OR	95% CI	OR	95% CI	OR	95% CI	OR	95% CI
SES	1.15*⁣*^*∗∗∗*^	[1.08, 1.23]	1.16*⁣*^*∗*^	[1.01, 1.32]	1.16*⁣*^*∗∗∗*^	[1.08, 1.24]	1.15	[1.00, 1.32]
Gender	1.22*⁣*^*∗∗∗*^	[1.17. 1.26]	1.63*⁣*^*∗∗∗*^	[1.51, 1.77]	1.22*⁣*^*∗∗∗*^	[1.18, 1.27]	1.55*⁣*^*∗∗∗*^	[1.42, 1.69]
Age	1.48*⁣*^*∗∗∗*^	[1.43, 1.54]	1.55*⁣*^*∗∗∗*^	[1.45, 1.67]	1.34*⁣*^*∗∗∗*^	[1.29, 1.39]	1.33*⁣*^*∗∗∗*^	[1.23, 1.44]
T2 IS	—	—	—	—	1.94*⁣*^*∗∗∗*^	[1.81, 2.07]	4.04*⁣*^*∗∗∗*^	[3.60, 4.54]
T2 AS	—	—	—	—	1.36*⁣*^*∗∗∗*^	[1.28, 1.45]	1.40*⁣*^*∗∗∗*^	[1.25, 1.56]
T2 RE	0.48*⁣*^*∗∗∗*^	[0.46, 0.50]	0.31*⁣*^*∗∗∗*^	[0.29, 0.34]	0.65*⁣*^*∗∗∗*^	[0.63, 0.68]	0.45*⁣*^*∗∗∗*^	[0.41, 0.49]

Abbreviations: AS, academic stress; IS, interpersonal stress; RE, resilience.

*⁣* ^*∗*^ *p* < 0.05; *⁣*^*∗∗*^*p* < 0.01; *⁣*^*∗∗∗*^*p* < 0.001.

**Table 6:** Multinomial logistic regression of T1 predictors on profiles' transition from T1 to T2.

T1 predictors	LP			CMP			CHP		
	OR	SE	95% CI	OR	SE	95% CI	OR	SE	95% CI
IS									
LP	REF	—	—	1.98*⁣*^*∗∗∗*^	0.05	[1.80, 2.17]	3.11*⁣*^*∗∗∗*^	0.12	[2.48, 3.89]
CMP	0.59*⁣*^*∗∗∗*^	0.06	[0.52, 0.66]	REF	—	—	1.83*⁣*^*∗∗∗*^	0.06	[1.54, 2.16]
CHP	0.27*⁣*^*∗∗∗*^	0.17	[0.19, 0.37]	0.53*⁣*^*∗∗∗*^	0.11	[0.43, 0.66]	REF	—	—
AS									
LP	REF	—	—	1.23*⁣*^*∗∗∗*^	0.05	[1.13, 1.35]	1.36*⁣*^*∗∗*^	0.11	[1.10, 1.68]
CMP	0.67*⁣*^*∗∗∗*^	0.06	[0.60, 0.75]	REF	—	—	1.13	0.08	[0.97, 1.33]
CHP	0.76	0.16	[0.55, 1.04]	0.98	0.11	[0.79, 1.20]	REF	—	—
RE									
LP	REF	—	—	0.77*⁣*^*∗∗∗*^	0.03	[0.73, 0.82]	0.91	0.08	[0.77, 1.07]
CMP	1.06	0.05	[0.97, 1.15]	REF	—	—	0.91	0.07	[0.79, 1.05]
CHP	1.04	0.12	[0.82, 1.33]	1.01	0.10	[0.83, 1.22]	REF	—	—

Abbreviations: AS, academic stress; CHP, co-occurring high profile; CMP, co-occurring moderate profile; IS, interpersonal stress; LP, low profile; RE, resilience; REF, reference group.

*⁣* ^*∗*^ *p* < 0.05; *⁣*^*∗∗*^*p* < 0.01; *⁣*^*∗∗∗*^*p* < 0.001.

**Table 7:** Multinomial logistic regression of T1 predictors on profiles' transition from T1 to T3.

T1 predictors	LP			CMP			CHP		
	OR	SE	95% CI	OR	SE	95% CI	OR	SE	95% CI
IS									
LP	REF	—	—	1.47*⁣*^*∗∗∗*^	0.04	[1.35, 1.60]	1.84*⁣*^*∗∗∗*^	0.11	[1.47, 2.29]
CMP	0.77*⁣*^*∗∗∗*^	0.05	[0.69, 0.86]	REF	—	—	1.38*⁣*^*∗∗∗*^	0.09	[1.17, 1.63]
CHP	0.65*⁣*^*∗∗*^	0.13	[0.50, 0.84]	0.79*⁣*^*∗*^	0.11	[0.64, 0.97]	REF	—	—
AS									
LP	REF	—	—	1.15*⁣*^*∗∗∗*^	0.04	[1.06, 1.25]	1.25*⁣*^*∗*^	0.11	[1.01, 1.56]
CMP	0.81*⁣*^*∗∗∗*^	0.05	[0.73, 0.90]	REF	—	—	1.20*⁣*^*∗*^	0.08	[1.03, 1.41]
CHP	0.79	0.14	[0.61, 1.04]	0.98	0.11	[0.80, 1.21]	REF	—	—
RE									
LP	REF	—	—	0.83*⁣*^*∗∗∗*^	0.03	[0.78, 0.87]	0.93	0.08	[0.79, 1.08]
CMP	1.00	0.04	[0.92, 1.09]	REF	—	—	0.85*⁣*^*∗*^	0.07	[0.74, 0.97]
CHP	1.10	0.11	[0.89, 1.37]	1.11	0.09	[0.92, 1.34]	REF	—	—

Abbreviations: AS, academic stress; CHP, co-occurring high profile; CMP, co-occurring moderate profile; IS, interpersonal stress; LP, low profile; RE, resilience; REF, reference group.

*⁣* ^*∗*^ *p* < 0.05; *⁣*^*∗∗*^*p* < 0.01; *⁣*^*∗∗∗*^*p* < 0.001

**Table 8:** Multinomial logistic regression of T2 predictors on profiles' transition from T2 to T3.

T2 predictors	LP			CMP			CHP		
	OR	SE	95% CI	OR	SE	95% CI	OR	SE	95% CI
IS									
LP	REF	—	—	1.67*⁣*^*∗∗∗*^	0.05	[1.51, 1.84]	3.83*⁣*^*∗∗∗*^	0.13	[2.99, 4.90]
CMP	0.54*⁣*^*∗∗∗*^	0.06	[0.48, 0.61]	REF	—	—	2.04*⁣*^*∗∗∗*^	0.09	[1.72, 2.43]
CHP	0.22*⁣*^*∗∗∗*^	0.19	[0.15, 0.32]	0.57*⁣*^*∗∗∗*^	0.11	[0.46, 0.70]	REF	—	—
AS									
LP	REF	—	—	1.43*⁣*^*∗∗∗*^	0.05	[1.30, 1.57]	1.28*⁣*^*∗*^	0.12	[1.01, 1.62]
CMP	0.77*⁣*^*∗∗∗*^	0.06	[0.69, 0.87]	REF	—	—	1.02	0.08	[0.87, 1.21]
CHP	0.72	0.17	[0.51, 1.01]	0.85	0.10	[0.70, 1.04]	REF	—	—
RE									
LP	REF	—	—	0.85*⁣*^*∗∗∗*^	0.03	[0.80, 0.90]	0.76*⁣*^*∗∗*^	0.09	[0.64, 0.91]
CMP	1.05	0.04	[0.96, 1.13]	REF	—	—	0.77*⁣*^*∗∗∗*^	0.08	[0.66, 0.90]
CHP	1.02	0.13	[0.79, 1.32]	1.23*⁣*^*∗*^	0.09	[1.03, 1.48]	REF	—	—

Abbreviations: AS, academic stress; CHP, co-occurring high profile; CMP, co-occurring moderate profile; IS, interpersonal stress; LP, low profile; RE, resilience; REF, reference group.

*⁣* ^*∗*^ *p* < 0.05; *⁣*^*∗∗*^*p* < 0.01; *⁣*^*∗∗∗*^*p* < 0.001.

## Data Availability

The data that support the findings of this study are available from the corresponding author upon reasonable request.
